# Organic carbon accumulation and aggregate formation in soils under organic and inorganic fertilizer management practices in a rice–wheat cropping system

**DOI:** 10.1038/s41598-023-30541-y

**Published:** 2023-03-04

**Authors:** Zhanhui Zhao, Yanli Mao, Songfeng Gao, Chunyang Lu, Chuanjiao Pan, Xiaoyu Li

**Affiliations:** 1 School of Surveying and Urban Spatial Information, Henan University of Urban Construction, Pingdingshan, 467036 People’s Republic of China; 2 School of Municipal and Environmental Engineering, Henan University of Urban Construction, Pingdingshan, 467036 People’s Republic of China; 3 State Experimental Station of Agro–Ecosystem in Fengqiu, State Key Laboratory of Soil and Sustainable Agriculture, Institute of Soil Science, Chinese Academy of Sciences, Nanjing, 210008 People’s Republic of China; 4 College of Resources and Environment, Shanxi Agricultural University, Taigu, 030801 People’s Republic of China

**Keywords:** Agroecology, Sustainability

## Abstract

Soil organic carbon (C) and aggregates are the important components of soil fertility and the foundation of sustainable agriculture. The storage and protection of SOC in aggregates is widely regarded as the material basis of soil organic C accumulation. However, current understanding of soil aggregate and its associated organic C is insufficient to elucidate the regulation mechanism of soil organic C. A nine-year field experiment including chemical fertilizer (FR) and organic manure (OM) treatments was set up in the eastern plain of Funiu Mountain, central China. Using chemical analysis, physical sieving as well as nuclear magnetic resonance (NMR) methods, we mainly probed into the response of soil organic C concentration and composition, and C functional groups, water-stable aggregates to different treatments. Furthermore, scanning electronic microscopy (SEM) and partial least square structural equation modelling (PLS-SEM) was conducted to characterise the different size aggregates and to analyse the mechanism of soil organic C accumulation and stabilisation at aggregate scales. After nine years of farming, OM treatment substantially increased soil organic C content (by 3.77 g kg^−1^) and significantly enhanced the formation of macro-aggregates (> 250 μm), while FR had no significant influence on soil organic C. At the aggregate scale, the amounts of soil organic C, C physical fractions (particulate and mineral-associated organic C), total nitrogen and microbial biomass carbon associated in macro-aggregates (> 250 μm) were obviously higher than that in micro-aggregates and silt + clay fraction, and OM treatment greatly increased the accumulation of soil organic C and its components in macro-aggregates. Moreover, microbial biomass carbon (MBC) amounts in aggregates were remarkably increased (27–116%) by the application of OM. And MBC had a positively effect on the physical fractions of SOC but not on the C chemical structure within aggregates. The present study indicated that soil organic C accumulation mainly rely on macro-aggregates (> 250 μm). Intra–particulate organic carbon (POC) and mineral-associated organic carbon (MOC) within macro-aggregates played an important role in soil organic C accumulation. Meanwhile, soil microbes were a driving force for the accumulation of soil organic C physical fractions (POC and MOC). We concluded that OM treatment accelerated the synergistic process between organic C sequestration and soil aggregation, and showed great potential to increase soil organic C accumulation.

## Introduction

Soil organic C is the key driver that promotes soil development and improves soil function^1^. Soil aggregates, the basic unit of soil physical structures, are important sites for circulating substances and energy in the soil, directly affecting the transformation and sequestration of soil organic C^2,3^. Some studies have revealed that soil clay adsorbs on the surface of organic matter rather than organic matter adsorbs on the surface of clay, and the encrusting of soil clay can greatly improve the stability of soil organic matter^4–6^. The static stability of soil organic C depends on the physical protection of aggregates (physical isolation), and the dynamic stability of soil organic C is driven by microorganisms (mineralized decomposition and assimilative accumulation), which often occur simultaneously and have a complex correlation^7,8^. However, as the key driver of soil organic C, soil microorganism distribution has high spatial heterogeneity owing to the restriction of the soil structure and microenvironment. This spatial heterogeneity significantly affects the biological stability of soil organic C^9^. Therefore, knowledge of the relationships between soil organic C and microbial biomass traits at the aggregate scale is crucial for understanding the biophysical mechanisms of soil organic C accumulation and protection.

Soil particle size fractionation combined with chemical and spectroscopic analyses is the most widely used method to study soil organic C dynamics within soil aggregates^3,10,11^. Soil organic C content and its components are deeply influenced by fertilisation because organic and inorganic fertilizer applications can quickly change the composition and quantity of soil organic C by adding C directly and increasing crop residue input indirectly^9,12,13^. Some past research revealed that applying organic manure increases soil activity and intermediate C pools more rapidly than mineral fertilizer^8,14,15^; for instance, microbial biomass carbon (MBC), an important soil active C pool component that reflects soil carbon-driving microbial activity, is easily influenced by C and nitrogen in organic and inorganic fertilizers. Particulate organic carbon (POC), the organic–mineral complex of newly formed soil organic C cemented with sand particles (53–2000 μm), belongs to the intermediate C pool and is regarded as a short-term plant nutrition pool. These relatively active C components are sensitive to exogenous C inputs in topsoil, such as organic manure and plant residues, and provide an early indicator of C dynamics and soil organic C changes^16,17^. In contrast, mineral-associated organic carbon (MOC) is tightly adsorbed by soil silt and clay (< 53 μm). The long persistence of MOC is responsible for long-term soil organic C sequestration in bulk soil owing to its high contribution to total soil organic C, making this fraction particularly important in soil C sequestration^18^. However, owing to the strong mineral and aggregate protection for MOC, inherent variation of MOC, and small change relative to the total soil organic C pool, it is often difficult for the MOC content to respond quickly to fertilisation^9^.

Anthropogenic management affects the levels of recalcitrant organic C components characterised by organic C chemical structure. The preferential accumulation of organic C functional groups in aggregates depends on the aggregate size. For example, organic fertilizer application promotes O–alkyl C and di–O–alkyl C accumulation primarily in macro-aggregates^19^; in contrast, organic manure amendment may decrease the O–alkyl–C and alkyl C content from macro-aggregate to silt–clay fractions^15^. Moreover, soil organic C chemical composition is primarily determined by the interaction between the organisms responsible for decomposition and the mineral soil matrix and not the nature of substrate input^14^. The knowledge of C functional groups is an effective way to explore soil organic C physical fraction accumulation mechanisms^20–22^. In addition, the key structural components of soil organic matter are largely composed of macromolecular inputs from degrading biomass^23^. The accumulation of microbial residues and products are the most important factor influencing aromatic organic molecules bound to clay-associated organic matter, depending on the microbial biomass^24^. The microbial decomposition of organic molecules is related to the distribution and spatial isolation of organic molecules in aggregates^1,4^. Therefore, both the soil organic C physical fractions and the chemical structure turnover and sequestration are presumably closely associated with microbial activity. Based on these reports, the stabilization mechanisms of soil organic C are closely related to interactions between the physicochemical characteristics of organic molecules and microbial activity. The compartmental retention and isolation of organic microbes within aggregates may be a key link in the soil organic C sequestration mechanism.

In the past decades, several long-term field experiments monitoring the dynamic variation in soil organic C in central China have confirmed that organic amendment application improves soil macro-aggregate structure and physically protects SOM from biodegradation by microorganisms^8,9,19,25^. However, the relationships among the structural characteristics of soil organic C physicochemical components, microbial biomass, and soil organic C accumulation occurring at aggregate scales after organic amendment application remain unclear. Further investigation is necessary to explore the mechanisms of organic component accumulation in aggregates via diverse spectral analyses combined with traditional physicochemical fractionation analysis of soil organic C. In this study, physical fractionation procedures were used to separate the spatial arrangement of primary and secondary organic–mineral particles based on the density and size of the primary aggregate using the procedure described by Six^26^. Advanced analytical techniques, such as nuclear magnetic resonance (NMR) spectroscopy and scanning electronic microscopy (SEM), were applied to characterise the organic C components in aggregates. The objectives of the current study were (1) to investigate the effects of fertilisation on soil organic C physical fractions, functional chemical components, and MBC within aggregates and (2) to understand the mechanism of soil organic C accumulation and stabilisation at aggregate scales. We hypothesised that soil organic C quantity and physicochemical component characteristics would be affected by the compartmentalisation, isolation, and stabilisation of soil organic C in aggregates of different sizes.

## Materials and methods

### Field experiment

The study was conducted in an experimental demonstration field (33°42′N, 113°10′E) situated in the eastern plain of Funiu Mountain, central China. The experiment began in October 2010 in a well-drained field in a typical paddy-upland rotation region with medium soil fertility. The area was an interlaced warm temperate zone with a subtropical climate, with a mean annual temperature and rainfall of 15 °C and 1000 mm, respectively, and 214–231 frost-free days annually. Before the commencement of the experiment, the soil in the plough layer (0–20 cm) had a bulk density of 1.33 g cm^−3^, containing 27.1% clay, 29.5% silt, and 43.4% sand, with 9.28 g kg^−1^ organic C, 890 mg kg^−1^ total nitrogen, 310 mg kg^−1^ total phosphorus, 17,260 mg kg^−1^ total potassium, 17.6 cmol kg^−1^ cationic exchange capacity, and soil pH (soil: H_2_O ratio 1:2.5) 6.8. The soil type was cinnamon soil.

The experiment included the following two crops per year: (1) rice (*Oryza sativa* L.) grown from June to October and (2) wheat (*Triticum aestivum* L.) grown from October to May. The soil was tilled to a depth of 20 cm from the surface during the rice season but not during the wheat season. The experiment included two treatments: chemical fertilizer (FR; 225 kg N ha^−1^ + 90 kg P_2_O_5_ ha^−1^ + 90 kg K_2_O ha^−1^) and organic manure (OM; the amount of nitrogen fertilizer was replaced with organic manure, and the insufficient P_2_O_5_ and K_2_O content from organic fertilizer were supplemented with chemical fertilizer until it was the same as FR). OM, phosphorus (P_2_O_5_), and potassium (K_2_O) fertilizers were applied into the soil before sowing, and chemical nitrogenous fertilizer was applied at the rates of 40% and 60% of the total amount before sowing and during the elongation stage, respectively. Crops were harvested mechanically after manual sampling at maturity. After harvest, all crop residues were removed from all treated plots. The experiment was a randomized block design with three replicate plots per treatment. The area of each plot was 100 m^2^ (10 m × 10 m).

### Soil sampling and aggregate fractionation

Three intact soil cores (0–20 cm) from each subplot were collected using a 12 cm wide sampling stainless steel shovel and mixed to form one composite sample on 30 October 2019. The length of the sampling stainless steel shovel was 25 cm and its handle was about 1.5 m. Moist samples were immediately transferred to the laboratory in plastic boxes. Pre-soil samples were collected for testing in June 2010.

Moist soil samples were gently broken apart along natural breakpoints and passed through a 10-mm sieve. Plants and organic debris in the sieved soil were carefully identified and removed using forceps. After mixing thoroughly, a subsample of the 10-mm sieved soil was passed through a 2-mm sieve and then air-dried indoors. The air-dried soil samples were passed through a 0.15-mm sieve and then used to determine the total soil organic C content and C functional groups. The remaining subsample of the 10-mm sieved soil was air-dried and used for soil fractionation analyses, and four classes of aggregates (> 2000, 250–2000, 53–250, and < 53 μm) were obtained. After aggregate separation, appropriate soil samples of the aggregate fractions were separated to determine soil organic C and C functional groups.

### Soil aggregation and analysis

The physical fractions of soil aggregates were determined according to Elliott^27^. A sample of 100 g of each first air-dried subsample mentioned earlier was immersed in deionized water on top of a 2000 μm sieve. After 5 min of slaking, the sieve was manually moved up and down 60 times through a distance of 3 cm for 2 min. Water plus soil < 2000 μm was poured through a 250 μm sieve and a 53 μm sieve. The fractions remaining on 2000, 250, and 53 μm sieves were collected. Soil with particles < 53 μm was allowed to settle and centrifuged. Therefore, four aggregate size classes were obtained: (1) large macro-aggregate (> 2000 μm), (2) small macro-aggregate (250–2000 μm), (3) micro-aggregate (53–250 μm), and (4) silt + clay fraction (< 53 μm). Thereafter, the aggregate fractions were dried at 40 °C for soil organic C analysis. Soil organic C physical fractions was analysed using the density fractionation method for further fractionating macro-aggregates and micro-aggregates into different sub-fractions. Macro-aggregates (> 250 μm) were fractionated into coarse intra–particulate organic carbon (iPOC, > 250 μm), fine iPOC (53–250 μm), and silt + clay sub-fraction (MSC, < 53 μm). In addition, micro-aggregates (< 250 μm) were fractionated into the fine iPOC (53–250 μm) and silt + clay sub-fraction (mSC, < 53 μm). Fifty grams of macro- or microaggregates were placed into 250-ml centrifuge tubes, and 150 ml of 1.85 g cm^−3^ ZnBr solution was added. The tubes were inverted 10 times. These samples were centrifuged for 30 min at 4000 rpm after standing for 20 min. After centrifugation, the pellets remaining in the centrifuge tubes were washed with deionized water 3 times and resuspended in 150 ml of sodium hexametaphosphate (0.5%, w/v) solution, and the tubes were shaken for 18 h to completely disperse aggregates. Finally, the samples were sieved through 250- and 53-μm sieves to obtain coarse iPOC, fine iPOC, and silt + clay subfractions (MSC)^28^.

### Analysis of chemical properties

Soil chemical properties were measured in bulk soils and all aggregate fractions. The concentrations of soil organic C in bulk soil and aggregates were determined using potassium dichromate redox titration method^29^, and the total nitrogen (TN) was measured by Kjeldahl methods^30^. The MBC was measured using a TOC analyzer (Vario Max Elemental Analyzer)^31^.

### NMR spectroscopy

Solid-state cross-polarisation magic angle spinning (CPMAS) ^13^C NMR spectroscopy of soil organic matter was performed according to Zhang et al.^32^. First, the bulk soils and aggregates were treated with hydrofluoric acid (HF) solution to eliminate paramagnetic compounds for improving their sensitivity of solid-state ^13^C NMR spectroscopic analyses. For each sample, finely ground (< 0.2 mm) soil (3 g) was extracted with nine successive 50 ml aliquots of 2% HF, shaken for 1 h (five times), 16 h (three times), and 64 h (once), in that order^33^. Between treatments, the samples were centrifuged for 10 min at 4000 rpm, and the supernatant was discarded and replaced with a fresh 10% HF solution. After successive treatments, the soil sample was washed with deionized water to obtain pH 6–7 and then air-dried. ^13^C NMR analyses were performed using a Bruker Avance III HD 400 spectrophotometer at 100.6 MHz (400.13 MHz 1H frequency; Bruker BioSpin Corporation, Switzerland).

### Calculations and statistical analyses

The amount of organic C and TN in aggregates was calculated as follows:

$${\text{C}}_{{\text{amount - fraction}}} = {\text{C}}_{{\text{con - fraction}}} \times {\text{M}}_{{{\text{fraction}}}}$$

$${\text{N}}_{{\text{amount - fraction}}} = {\text{N}}_{{\text{con - fraction}}} \times {\text{M}}_{{{\text{fraction}}}}$$

where C_amount–fraction_ (g C kg^−1^ soil) and N_amount–fraction_ (g N kg^−1^ soil) are the amounts of organic C and TN in aggregates, C_con–fraction_ (g C kg^−1^ fraction) and N_con–fraction_ (g N kg^−1^ fraction) are the organic C and TN contents of aggregates, respectively. And M_fraction_ is the mass proportion of aggregates in the whole soil.

The differences in soil properties and soil organic C fractions among treatments were examined using one-way ANOVA with honest significant difference (HSD) as the post-hoc test. Differences at *p* < 0.05 were considered statistically significant. A stepwise method was used in linear regressions to identify the relationships between C functional group contents and C amounts in physical sub-fractions in macro-aggregates using SPSS 22.0 software package for Windows (SPSS Inc., IL, USA).

Partial least square structural equation modelling (PLS-SEM) was conducted to analyse the effects and effect sizes of fertilisation, soil aggregates and biomass on soil organic C and across the different soil organic C components. PLS-SEM analysis was performed using SmartPLS v3.2.7 Pro software^34^, and the key criteria of the overall coefficient of determination (*R*^2^), effect size (*f*^*2*^), and significance of the weights and loadings of the manifest variables were used to assess the structural model^35^. The cross-validated redundancy (*Q*^2^) was used to assess the predictive relevance of the model^36^.

### Ethical approval

Experimental research and field studies on cultivated crops and soils were all comply with local government, and international guidelines and legislation. The authors declare no competing interests.

## Results

### Aggregates and soil properties

**Figure 1:**
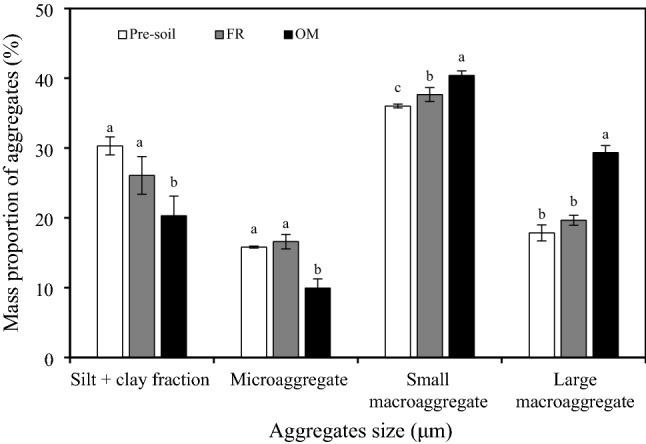
Mass proportion of aggregates in different treatments. Vertical bars denote the standard error of the mean (n = 3). Different letters a, b and c indicate significant differences between treatments for the same aggregate at *p* < 0.05. FR, chemical fertilizer; OM, organic manure.

The soil aggregate distribution was dominated by small macro-aggregates, whereas a relatively low proportion of the other fractions (silt + clay, micro-aggregates, and large macro-aggregates) was found (Fig. 1). Compared with pre-soil, the changes of the mass proportions of silt + clay and micro-aggregates in FR treatment were not significant, but OM treatment had the lowest mass proportions of silt + clay and micro-aggregates, which were lower by 33% and 37%, respectively. In contrast, the mass proportions of small and large macro-aggregates were increased by 5% and 10% in the FR treatment and by 12% and 64% in the OM treatment than in pre-soil, respectively (Fig. 1).

After nine years of fertilisation, the increase of soil organic C content in FR treatment was not significant compared with pre-soil. But, the soil organic C content in the OM treatment was significantly higher by 3.77 g kg^−1^ than that in pre-soil, respectively. TN and MBC contents also showed a similar trend; specifically, TN and MBC contents increased by 13% and 32% in the FR treatment and by 39% and 130% in the OM treatment compared with pre-soil, respectively (Table S1). Moreover, OM application significantly increased alkyl–C and the ratio of A/O–A (alkyl–C/O–alkyl C) but decreased aromatic–C and aromaticity index (AI) compared to pre-soil. However, FR had no substantial effect on C functional groups compared with pre-soil, and O–alkyl C and carbonyl–C showed no substantial differences across all treatments (Table S1).

### Aggregates associated organic C and TN

Compared with pre-soil, the organic C contents in micro-aggregates, small and large macro-aggregates of the FR treatment was significantly higher (by 26%, 20% and 34%), and the organic C contents in small and large macro-aggregates of the OM treatment also significantly increased by 66% and 194%, respectively (Table S2). Meanwhile, the amounts of aggregates associated TN in FR treatment had no significant change compared with pre-soil, but OM-treated soil had significantly lower (by 19%) TN amount in micro-aggregates and higher (by 45–116%) TN amount in small and large macro-aggregates than pre-soil.

The small and large macro-aggregates associated C:N ratio of FR and OM treatments presented higher (by 12–36%) values than pre-soil, and the OM treatment had the highest C:N ratio in micro-aggregates, small macro-aggregates, and large macro-aggregates followed by the FR treatment (Table S2). Moreover, MBC amounts in all aggregate classes were remarkably higher (27%–116%) in the OM treatment than in pre-soil. FR treatment only increased the MBC content in small and large macro-aggregates but had no significant effect on micro-aggregates (Table S2).

**Figure 2:**
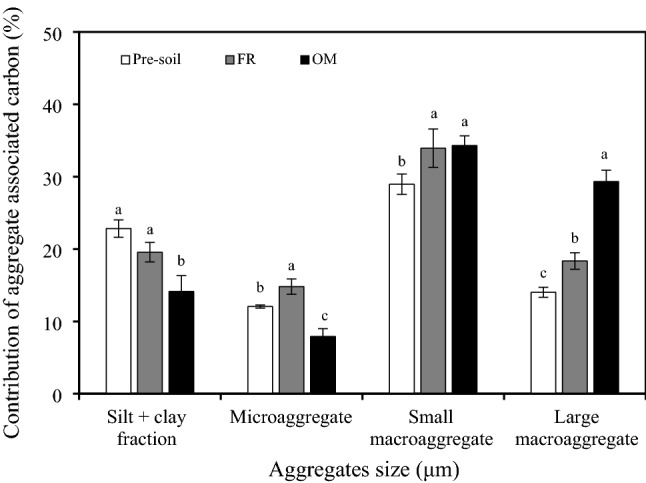
Contribution of the aggregates associated organic carbon amounts to 0-20 cm bulk soil in different treatments. Vertical bars denote the standard error of the mean (n = 3). Different letters a, b and c indicate significant differences between treatments for the same aggregate at *p* < 0.05. *FR* chemical fertilizer, *OM* organic manure.

In addition, the small macro-aggregates associated organic C contributed the most to organic C in 0–20 cm bulk soil across all treatments (Fig. 2). Compared with pre-soil, FR treatment increased the contribution of organic C associated in micro-aggregates, small macro-aggregates, and large macro-aggregates to the 0–20 cm bulk soil by 23%, 17%, and 31%, respectively. Meanwhile, the OM treatment decreased the contribution of organic C amounts in silt + clay and micro-aggregates by 38% and 34%, respectively, whereas that of small and large macro-aggregates increased by 19% and 109%, respectively, compared with pre-soil (Fig. 2).

### Soil organic C compositions

**Figure 3:**
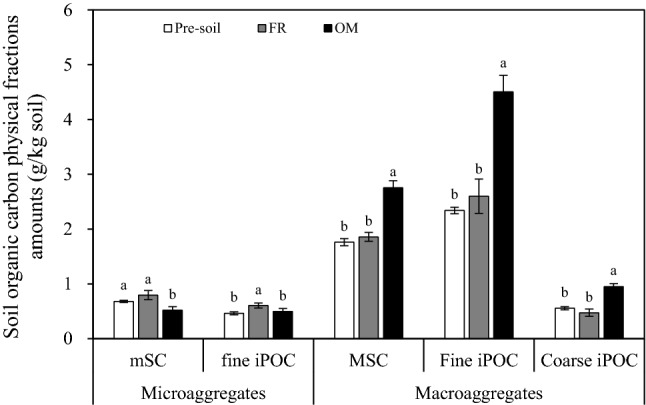
Soil organic carbon physical fractions amounts in different treatments. Vertical bars denote the standard error of the mean (n = 3). Different letters a, b and c indicate significant differences between treatments for the same physical fraction of soil organic carbon at *p* < 0.05. *FR* chemical fertilizer, *OM* organic manure, *mSC* mineral-incorporated organic carbon in micro-aggregates, *fine iPOC* fine intra-particulate organic carbon in micro-aggregates, *MSC* mineral-incorporated organic carbon in macro-aggregates, *Fine/coarse iPOC* fine/coarse intra-particulate organic carbon in macro-aggregates.

Compared with pre-soil, FR only significantly increased the amount of fine iPOC within micro-aggregates by 30.83%. In contrast, OM mainly boosted the physical fractions amounts of organic C within macro-aggregates, specifically of MSC, fine iPOC, and coarse iPOC (by 56.43%, 92.56%, and 70.45%, respectively), but decreased mSC by 23.80% (Fig. 3).

In bulk soil, the relative amounts of C functional groups in FR treatment shown no significant difference with pre-soil, but OM treatment had higher (by 35%) alkyl-C amount and lower (by 20%) aromatic-C than pre-soil. Similarly, A/O-A ratio and AI of OM-treated soil were significantly higher (by 39%) and lower (by 21%) than that in pre-soil (Table S1). At aggregate scales, only the relative amounts of small and large macro-aggregates associated O–alkyl C in FR treatment increased by 4% and 13% compared with pre-soil, respectively (Table S3). In the OM treatment, O–alkyl C, aromatic–C, and carbonyl–C within silt + clay fraction and alkyl–C, O–alkyl C, aromatic–C, and carbonyl–C within small micro-aggregates were significantly decreased, whereas alkyl–C, O–alkyl C, aromatic–C, and carbonyl–C within large macro-aggregates significantly increased compared with pre-soil (Table S3). Correspondingly, the contributions of O–alkyl C and aromatic–C within silt + clay fraction and alkyl–C and O–alkyl C within micro-aggregates to 0–20 cm bulk soil were significantly decreased in the OM treatment than in pre-soil. Meanwhile, the contributions of O–alkyl C, aromatic–C, and carbonyl–C within small and large macro-aggregates significantly increased (by 18%–80%, 48%–81%, 54%–60%, respectively) in the OM treatment than in pre-soil. Compared to pre-soil, OM-treated soil had a lower (by 27%) alkyl–C contribution within small macro-aggregates but a higher (by 25%) alkyl–C contribution within large macro-aggregates to 0–20 cm bulk soil (Table 1).

**Table 1:** Contribution of the aggregates associated C functional groups to 0-20 cm bulk soil in different treatments (%).

Aggregate size	Treatment	alkyl-C	O-alkyl C	aromatic-C	carbonyl-C
Silt and clay fraction (< 53 μm)	Pre-soil	39.12 ± 4.73a	28.43 ± 2.42a	27.62 ± 2.81a	29.79 ± 7.22a
	FR	29.04 ± 0.47a	23.65 ± 4.09ab	26.11 ± 3.86ab	38.06 ± 4.77a
	OM	21.77 ± 5.00a	19.30 ± 0.71b	20.34 ± 3.19b	22.70 ± 10.79a
Micro-aggregates (53–250 μm)	Pre-soil	20.77 ± 2.60a	15.03 ± 0.34a	14.21 ± 0.6ab	13.78 ± 4.04a
	FR	20.32 ± 1.26a	16.00 ± 0.98a	15.64 ± 2.88a	13.28 ± 2.02a
	OM	9.90 ± 1.64b	10.00 ± 1.56b	10.74 ± 2.32b	9.02 ± 2.39a
Small macro-aggregates (250–2000 μm)	Pre-soil	45.81 ± 4.77a	35.17 ± 2.93b	31.83 ± 3.3b	28.29 ± 2.55b
	FR	38.66 ± 4.22ab	36.46 ± 0.98b	40.21 ± 8.49ab	39.45 ± 7.28a
	OM	33.30 ± 4.56b	41.57 ± 2.33a	47.18 ± 1.04a	43.48 ± 4.76a
Large macro-aggregates (> 2000 μm)	Pre-soil	19.64 ± 1.64b	17.24 ± 1.3b	17.83 ± 1.07b	18.20 ± 4.16b
	FR	20.88 ± 0.90b	19.57 ± 0.78b	19.22 ± 2.65b	19.01 ± 3.81b
	OM	24.50 ± 0.32a	30.99 ± 1.35a	32.32 ± 3.16a	29.14 ± 1.72a

The results show means ± standard deviations (n = 3). Different letters at the same columns indicate significant differences (*p* < 0.05) between treatments.

*FR* chemical fertilizer, *OM* organic manure.

### Relationships between soil organic C compositions and aggregates

Regression analysis showed that the organic C content in the bulk soil was significantly, positively, and linearly correlated to the mass proportion of large macro-aggregates (*p* < 0.001), MSC (*p* < 0.001), alkyl–C (*p* < 0.01), and MBC content in micro-aggregates (*p* < 0.001) across the treatments (Table 2). Alkyl–C content in the bulk soil was significantly, positively, and linearly correlated with coarse iPOC (*p* < 0.001) and MBC (*p* < 0.01) within macro-aggregates. In contrast, aromatic–C content in the bulk soil was significantly, negatively, and linearly correlated with MSC (*p* < 0.01) and MBC (*p* < 0.01) within macro-aggregates (Table 2).

**Table 2:** Relationships among soil organic C, C functional group contents, organic C physical components and water-able aggregate.

Index	Stepwise regression equation	*R*^2^	Significance level
SOC	SOC = 3.07 + 0.338 × WLA	0.98	*p* < 0.001
	SOC = 2.713 + 0.696 × MSC	0.96	*p* < 0.001
	SOC = -0.645 + 0.513 × alkyl-C	0.85	*p* < 0.01
	SOC = 5.698 + 0.025 × MBC_micro_	0.98	*p* < 0.001
C functional group			
Alkyl-C	Alkyl-C = 6.569 + 1.595 × Coarse iPOC	0.9	*p* < 0.001
Aromatic-C	Aromatic-C = 25.453–0.695 × MSC	0.71	*p* < 0.01
Alkyl-C	Alkyl-C = 9.807 + 0.054 × MBC_macro-2_	0.83	*p* < 0.01
Aromatic-C	Aromatic-C = 23.764–0.024 × MBC_macro-1_	0.65	*p* < 0.01

*SOC* soil organic C in 0–20 cm bulk soil, *WLA* large macroaggreagate, *MSC* mineral-incorporated organic carbon in macro-aggregates, *coarse iPOC* coarse intra-particulate organic carbon in macro-aggregates, *MBC*_*micro*_ microbial biomass carbon within in micro-aggregate, *MBC*_*macro-1*_ microbial biomass carbon within in small macro-aggregate, *MBC*_*macro-2*_ microbial biomass carbon within in large macro-aggregate.

### Relationships between aggregates associated C and nitrogen

In terms of organic C physical fractions, aggregates associated organic C, TN, and MBC contents were significantly correlated with mSC, MSC, and fine and coarse iPOC. However, the organic C chemical compositions showed no significant correlation with organic C, TN, and MBC contents and organic C physical fractions in aggregates (Fig. S1).

**Figure 4:**
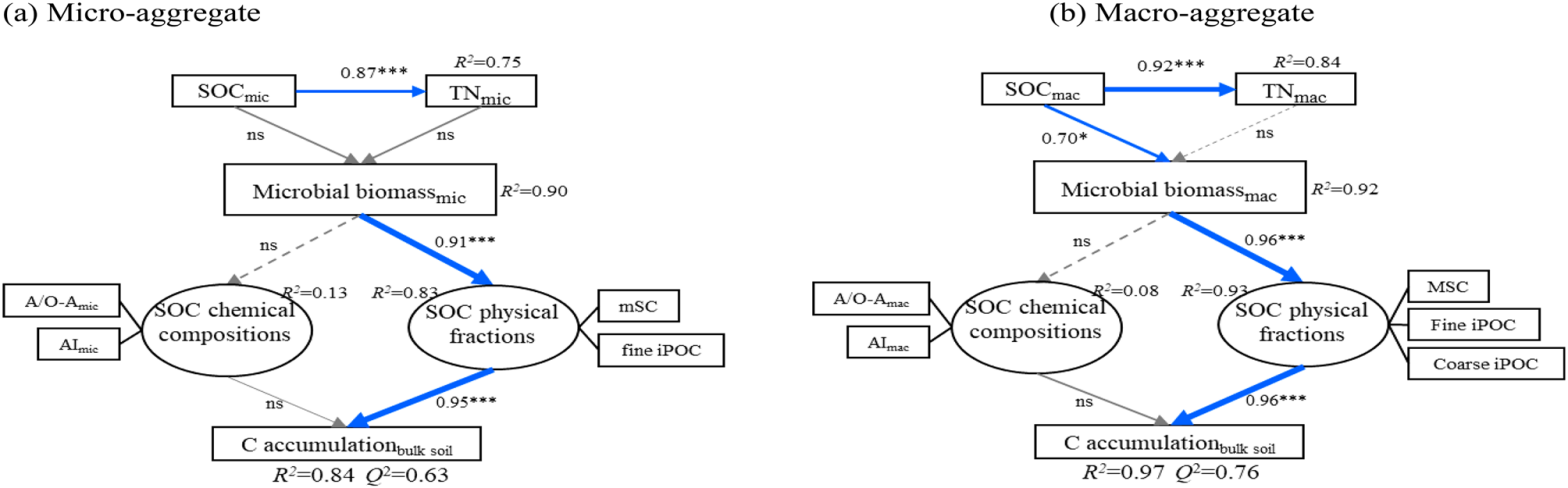
Partial least square structural equation model (PLS-SEM) showing the relationships among soil chemical properties, soil organic C chemical composition and organic C physical components in micro-aggregates (**a**), macro-aggregates (**b**). The overall coefficient of determination (*R*^2^), the effect size (*f*^2^), and the significance of the weights and loadings of manifest variables were used to assess the structural model. Solid lines and dashed lines indicate positive and negative correlations, respectively. The arrow width is proportional to the strength of the path coefficients. ns indicates nonsignificant relationships, while ** and *** indicate significance at *p* < 0.01 and 0.001, respectively. *SOC* soil organic C, *TN* total nitrogen, *MBC* microbial biomass carbon, *mSC* mineral-incorporated organic carbon in micro-aggregates, *fine iPOC* fine intra-particulate organic carbon in micro-aggregates, *MSC* mineral-incorporated organic carbon in macro-aggregates, *Fine/coarse iPOC* fine/coarse intra-particulate organic carbon in macro-aggregates. The subscript letters _mic_ and _mac_ represented soil properties of microaggregates and macroaggregates, respectively.

At the micro-aggregate scale, the micro-aggregate associated organic C was significantly correlated with TN (*p* < 0.001); however, microbial biomass was not directly affected by organic C and TN within micro-aggregate. Moreover, microbial biomass directly affected the organic C physical fractions (including mSC and fine iPOC) and further promoted the organic C accumulation in bulk soil but had no significant effect on the chemical composition of organic C (Fig. 4a). Within the macro-aggregate fractions, TN was also closely related to organic C; however, only organic C directly affected MBC, which further influenced organic C physical fractions (including mSC and fine iPOC), increasing organic C accumulation in bulk soil. Throughout the entire pathway effects of organic C, MBC, and organic C physical components within macro-aggregates and organic C accumulation in bulk soil, the chemical composition of organic C showed no significant correlation with the above index components (Fig. 4b).

## Discussion

### Variation characteristics of organic C in aggregates

It is well known that is aggregates the foundation of soil structure. Soil aggregation is closely correlated with organic C content in bulk soil and depends on aggregate size^2,4^. In this study, nine years of OM treatment greatly increased the quantity of macro-aggregate. Our results also suggested that organic C was significantly correlated with macro-aggregates (Table 2), and macro-aggregates were the main contributors to organic C (Fig. 2). This result was possibly due to the organic C introduced into the soil by OM application, which directly increases the soil exogenous C and acts as an organic building block to promote aggregate formation^13,14^. Therefore, the OM application improved the organic C content and significantly increased the number of macro-aggregates. Some researchers revealed that organic C accumulation is mainly rely on the increase in POC and the enhancement of physical protection for organic C; in contrast, the old organic C with a higher degradation degree and smaller volume is stored in silt and clay particles^2,4,37^. In the present research, macro-aggregate-associated physical fractions (MSC, fine and coarse iPOC) were significantly increased by OM treatment, whereas no significant change was observed in the FR treatment (Fig. 3), possibly because OM introduces more organic matter into the soil than FR, increasing soil C source and providing key building blocks for the accumulation of physical components. In addition, organic C physical fractions were higher within macro-aggregates than within micro-aggregates (Fig. 3), suggesting that macro-aggregates and not micro-aggregates or silt and clay fractions were the main sites of C accumulation.

In addition, the stability and sequestration time of organic C also depended on its chemical composition and the resistance of C molecular structure to degradation^4^. Some researchers had reported organic amendments can easily improve degradable C functional group (e.g., carbohydrates) accumulation in soil^9,12^. However, in this study, macromolecular C compounds with a stable chemical structure were significantly changed by fertilisation. In particular, fertilisation (including OM and FR) significantly increased alkyl–C relative amounts, indicating the presence of long-chain aliphatic compounds, waxes, and cutins, as well as A/O–A, but decreased aromatic–C relative amounts, indicating the presence of lignin and AI in bulk soil (OM treatment) (Table S1). What’s more, both of alkyl–C, O–alkyl–C, aromatic–C and carbonyl–C tended to accumulate in macro-aggregates (OM treatment), possibly because fertilisation increased organic residue input, such as increased root biomass with FR and increased organic matter from OM^1^. Some scholars had revealed that carbohydrates and proteins in organic residues were decomposed first in soil, whereas some hard-to-decompose components (such as alkyl–C and aromatic–C) were enriched in soil, which were further decomposed and transformed by microorganisms into hard-to-decompose humus that exists for a long time^4,38^. In addition, the study had confirmed that macro-aggregates were the main C storage sites (Fig. 2 and Table S2). And macro-aggregates could accommodate C molecules with different degrees of decomposition, including different types of C functional groups (Table 1 and Table S3). Therefore, it’s could be further deduced the macro-aggregates associated C contained both the old C originally accumulated in soil and the new C imported from foreign soil because the simultaneous presence of C compounds with different chemical structures.

Moreover, previous studies have shown that the adsorption of clay particles and the storage of aggregates are the dominant mechanisms of the stability of degraded soil organic residues^4,6,39^. Therefore, soil clay was treated as the main site for long-term storage of older C with higher stability because it is less vulnerable to persecution and longer turnaround times. Different from previous studies, soil organic C components (including C functional groups and physical fractions) and MBC were mainly aggregated in the macro-aggregates (OM treatment). This might be due to that the larger size aggregate could contain more new organic matter than the smaller aggregate relying on its large space advantage though the mechanical stability of macro-aggregate was usually lower than clay mineral soil particles^3^. And Yudina et al.^40^ also suggested that macro-aggregates have a greater capacity for nutrients and biological activities than micro-aggregates or silt + clay particles. Thus, it’s could be speculated that the macro-aggregate might act as the main site for the degradation of organic matter and the occurrence of microbial proliferation.

### Linking organic C with microbial biomass within aggregates

Soil organic C and aggregates are interdependent communities; organic C acts as an organic binder and promotes the formation of soil aggregates, and the distribution and stability of aggregates affect the decomposition and transformation of organic C^2,3^. Similar to the principle of cooperative interaction between organic C and aggregates, the highest soil organic C content and remarkable changes in soil aggregate formation (i.e., the increase in the mass proportion of macro-aggregates) simultaneously occurred in the OM treatment. The most immediate reason for this result might be that OM amendment increased exogenous matter input, and the new added organic matter was adsorbed with soil particles into the aggregate. The increased soil organic C simultaneously acted as a cementing agent to promote the formation of macro-aggregates with the continuous application of exogenous OM. In addition to the natural adsorption of soil particles, microorganisms are another important factor influencing aggregate distribution. Previous study had proved that mycelia bind mineral particles and organic matter to form larger aggregates^41^. In the study, MBC in the macro-aggregates was much higher than that in the micro-aggregates with the increase of microbial activity in the soil (Tables S1, S2). This finding is consistent with the results of a previous study^42,43^.

**Figure 5:**
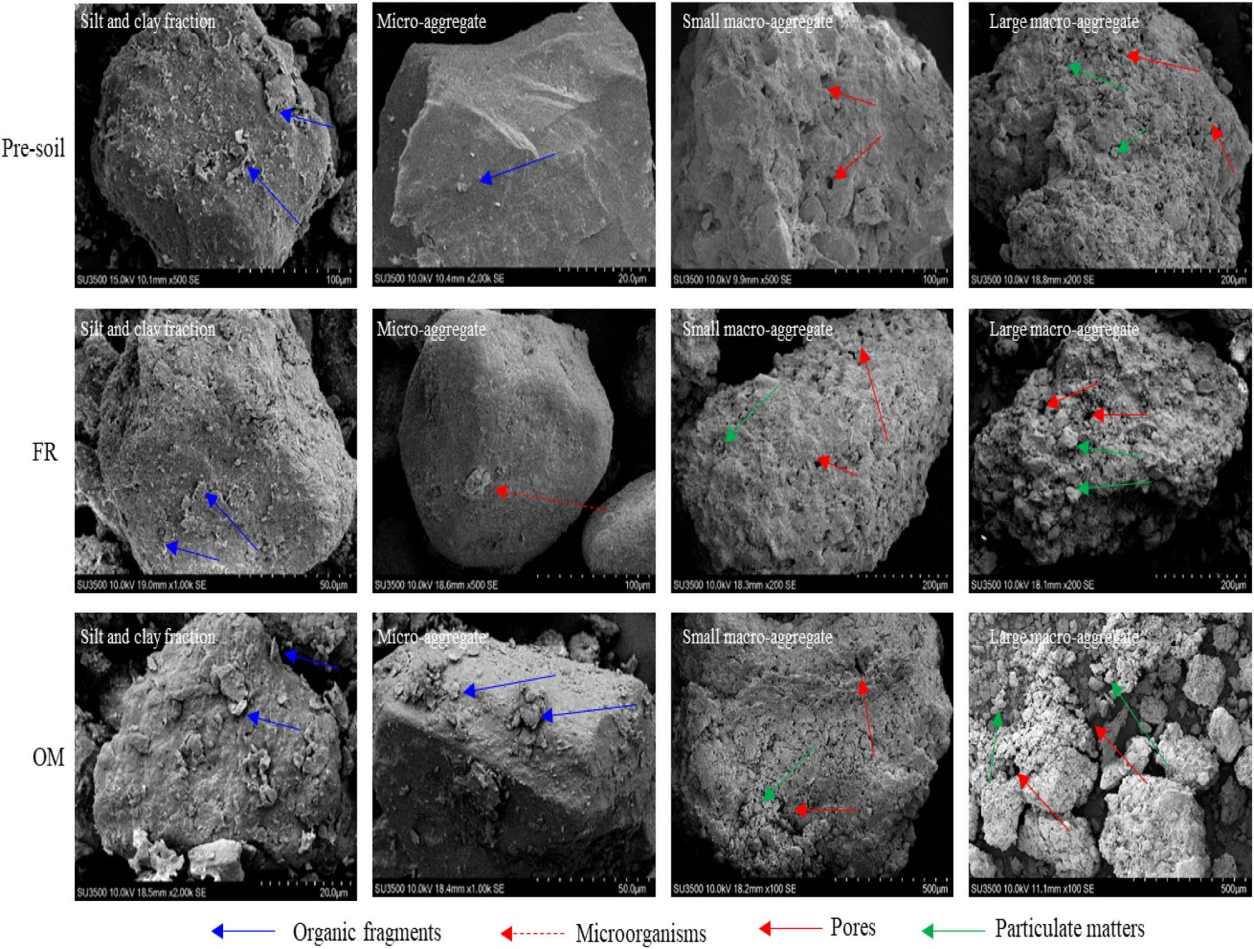
Scanning electron micrograph of water-able aggregates in different treatments.

The present study also found the increased MBC amount occurred simultaneously in all aggregate classes under OM treatment (Table S2). Concerning the variation in soil C and nitrogen contents, OM treatment also remarkably increased organic C and TN contents, which was equivalent to providing food and nutrition for soil microorganisms, respectively. Naturally, the microbial population increased rapidly, accelerating the contribution of available C sources in the soil to organic C accumulation in the form of metabolites^44,45^. The result of PLS-SEM analysis also cleared the relationship between microorganism biomass, aggregates associated organic C and nitrogen (Fig. 4). Microorganisms were unaffected by organic C content in micro-aggregates but were significantly directly affected in macro-aggregates (Fig. 4), possibly because of the spatial characteristics of the aggregates. The space occupied by micro-aggregates restricted the growth of microorganisms; meanwhile, the macro-aggregates had a relatively large space, but microorganism growth was restricted by C instead of space (Fig. 5). The scanning electron micrograph further proved the above deduction, which shown that the surface of the silt + clay fraction and micro-aggregates were smooth and seemingly hard, and the small and large macro-aggregates were loose, porous, and full of vitality (Fig. 5). In addition, the surfaces of silt + clay fraction and micro-aggregates treated with OM had more organic debris than those treated with FR or pre-soil, which might provide a C source for microorganisms. The macro-aggregates were seemly looser and had clearer soil particle textures; such structural features might be more conducive to microbial survival (Fig. 5). Therefore, all aggregates of different sizes were carriers of microbial survival under conditions of abundant nutrients, but macro-aggregates might possess more microbial biomass depending on their larger space and quantity.

**Figure 6:**
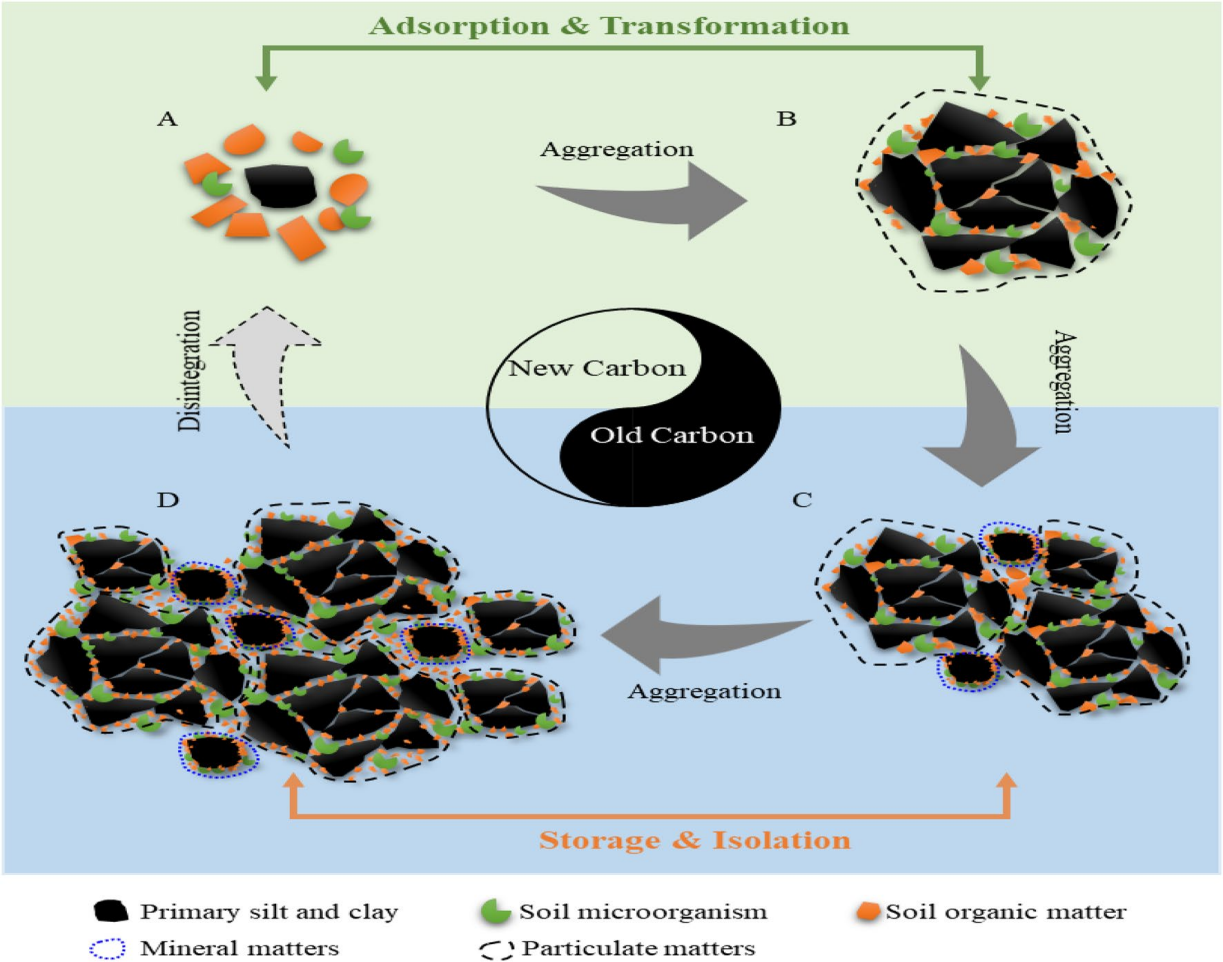
Schematic overview of soil aggregation process and organic C accumulation mechanisms in aggregates. (**A**–**D**) indicate silt + clay fraction (< 53 μm), micro-aggregate (53–250 μm), small macro-aggregate (250–2000 μm) and large macro-aggregate (> 2000 μm), respectively.

Moreover, the PLS-SEM analysis showed that MBC had a substantial effect on the physical fractions of organic C but not on the C chemical structure within micro-aggregates and macro-aggregates (Fig. 4). These results suggested that soil microbes were a driving force for the accumulation of organic C physical fractions (POC and MOC). By continuously adding exogenous organic matter (OM treatment), the stimulated microorganisms accelerated the decomposition and renewal of organic matter. The soil organic fragments digested by microorganisms were captured by soil clay minerals or aggregates to gradually form MOC or POC, forming primary soil grain units (possibly micro-aggregates). These primary soil grain units continuously aggregated to form larger size aggregates (Fig. 6). This aggregate-forming mechanism conforms to the aggregate hierarchy theory^6^. According to the characteristics of the electron microscope image, the silt + clay fractions and micro-aggregates were independent monomers, but the small and large macro-aggregates were soil complexes composed of multiple soil grain units (Fig. 5). Considering that the amounts of MOC and POC in larger size aggregates were much higher than those in smaller aggregates, it could be speculated that the composite structure of the macro-aggregates might be the hotbed of organic C physical fraction accumulation. However, the relationship between the structural characteristics of aggregates and organic C components accumulation requires further clarification in future studies.

Overall, OM remarkably promoted soil microbial biomass by affecting organic C more than TN within aggregates. Organic residues derived from microbial degradation were first adsorbed onto the silt + clay fractions, forming organic–inorganic complexes. Subsequently, the soil particles continuously coalesced to form progressively larger size aggregates. Most of the physically stable organic C fractions were isolated from macro-aggregates. Therefore, macro-aggregates are associated with more stable and older C particles, but smaller silt + clay fractions and micro-aggregates might capture younger C, depending on the time to capture C (Fig. 6). According to the redistribution theory of aggregates^46^, some of the old C associated with the macro-aggregates returned to the starting point of soil aggregation with macro-aggregate fragmentation and reflowed from small to large size aggregates. During the process of fragmentation and repolymerization, all aggregates of different sizes had the opportunity to contact the old and new C. The synchronous increase and decrease of aggregate-associated C functional groups with different stabilities also indirectly confirmed the existence of C with varying degradation grades in aggregates of all sizes (Table S3). Considering the characteristics of the electron microscope images and the distribution of organic C components in aggregates, different size aggregates exert different functions on C fixation. The silt + clay fraction and micro-aggregates captured and transformed exogenous C, and macro-aggregates mainly stored and isolated exogenous C owing to their larger space. Therefore, we concluded that aggregates of all sizes could capture both new and old C; however, macro-aggregates could store more C but had poor physical stability. Optimising fertilisation strategies (e.g., OM application) increased organic C sequestration by preferentially increasing the number of macro-aggregates.

## Conclusions

The present study quantified the variation of soil organic C content and water-stable aggregate under organic and inorganic fertilisation in a rice–wheat cropping system. The results indicated that OM application for nine years substantially increased soil organic C content, facilitated the formation of macro-aggregates, and enhanced synergistic promotion of aggregates associated organic C and macro-aggregates. The study found that organic C physical fractions (iPOC and silt + clay sub-fraction) and C functional groups with different stability were mainly accumulated in macro-aggregates. Scanning electron microscope photograph also revealed the structural advantages of macro-aggregates over silt + clay fraction and micro-aggregates in Cn sequestration. These results demonstrated that the silt + clay fraction and micro-aggregates mainly captured and transformed exogenous C, and the macro-aggregates mainly stored and isolated exogenous C owing to their larger space. By studying the responses of the quantity of soil organic C and aggregates to organic and inorganic fertilizer management practices, the organic amendment may be a feasible soil management strategy to improve soil organic C sequestration and soil aggregates structure for the paddy-upland rotation region in central China.

## Supplementary Information

Supplementary Information.

## Data Availability

The datasets used during the current study available from the corresponding author on reasonable request.

## Abbreviations

C: Carbon
MBC: Microbial biomass carbon
POC: Particulate organic carbon
MOC: Mineral-associated organic carbon
FR: Chemical fertilizer
OM: Organic manure
AI: Aromaticity index
iPOC: Intra-particulate organic carbon
MSC: Coarse silt + clay sub-fraction
mSC: Fine silt + clay sub-fraction
