# Supplementary material for: Organic carbon accumulation and aggregate formation in soils under organic and inorganic fertilizer management practices in a rice–wheat cropping system
Source: Sci Rep. 2023 Mar 4;13:3665. doi: 10.1038/s41598-023-30541-y (PMC9985631; doi:10.1038/s41598-023-30541-y)
Supplement: Supplementary file 1 — Supplementary Information. [file 41598_2023_30541_MOESM1_ESM.docx]

# Supplementary material:

Table S1 Soil chemical properties and the relative amounts (%) of different C functional groups in the different treatments.

| Treatment | SOC | TN | C:N ratio | MBC | alkyl-C | O-alkyl C | aromatic-C | carbonyl-C | A/O-A | AI |
| --- | --- | --- | --- | --- | --- | --- | --- | --- | --- | --- |
| Pre-soil | 9.28±0.26b | 0.89±0.03c | 10.4±0.65a | 137.16±6.48c | 19.23±1.8b | 54.08±0.82a | 19.24±0.42a | 7.46±1.29a | 0.36±0.04b | 20.8±0.74a |
| FR | 9.52±0.36b | 1.01±0.04b | 9.4±0.57a | 181.42±5.95b | 20.74±0.65b | 54.03±2.25a | 18.1±2.13ab | 7.13±1.32a | 0.39±0.01b | 18.99±2.36ab |
| OM | 13.05±0.48a | 1.24±0.05a | 10.53±0.46a | 316.12±8.46a | 25.9±1.36a | 51.79±2.55a | 15.35±1.41b | 6.96±1.82a | 0.5±0.03a | 16.52±1.82b |

The results show means ± standard deviations (n = 3). Different letters at the same columns indicate significant differences (*p* < 0.05) between treatments. SOC, soil organic C (g kg^-1^); TN, total nitrogen (g kg^-1^); MBC, microbial biomass carbon (mg kg^-1^); A/O-A, alkyl-C/O-alkyl C; AI, aromaticity index; FR, chemical fertilizer; OM, organic manure.

Table S2 SOC, TN, MBC amounts and C:N ratio for each water stable aggregate size in the different treatments.

| Item | Treatment | Silt + clay fraction  (<53 μm) | Micro-aggregate  (53–250 μm) | Small macro-aggregate  (250–2000 μm) | Large macro-aggregate  (>2000 μm) |
| --- | --- | --- | --- | --- | --- |
| SOC  (g/kg soil) | Pre-soil | 2.12±0.06a | 1.12±0.01b | 2.69±0.1c | 1.3±0.1c |
|  | FR | 1.86±0.09a | 1.41±0.12a | 3.23±0.31b | 1.75±0.12b |
|  | OM | 1.84±0.22a | 1.04±0.18b | 4.48±0.3a | 3.83±0.34a |
| TN  (g/kg soil) | Pre-soil | 0.26±0.01a | 0.13±0a | 0.32±0.01b | 0.16±0.01b |
|  | FR | 0.23±0.02a | 0.15±0.01a | 0.34±0.01b | 0.19±0b |
|  | OM | 0.21±0.04a | 0.11±0.02b | 0.46±0.04a | 0.34±0.03a |
| C:N ratio | Pre-soil | 8.29±0.13a | 8.3±0.04b | 8.52±0.22b | 8.29±0.22c |
|  | FR | 8.16±0.4a | 9.5±0.3ab | 9.5±0.64a | 9.37±0.51b |
|  | OM | 8.86±0.7a | 9.54±0.99a | 9.85±0.43a | 11.27±0.13a |
| MBC  (mg/kg soil) | Pre-soil | 36.53±2.27b | 23.31±1.34b | 66.38±2.51c | 33.05±1.98c |
|  | FR | 36.64±2.48b | 25.09±2.4ab | 93.15±5.49b | 38.42±2.17b |
|  | OM | 55.7±5.76a | 29.65±4.54a | 141.79±6.12a | 88.24±4.45a |

The results show means ± standard deviations (n = 3). Different letters at the same columns indicate significant differences (*p* < 0.05) between treatments. SOC, soil organic C; TN, total nitrogen; MBC, microbial biomass carbon; FR, chemical fertilizer; OM, organic manure.

Table S3 Relative amounts of C functional groups within aggregates in different treatments.

| Aggregate size | Treatment | alkyl-C | O-alkyl C | aromatic-C | carbonyl-C |
| --- | --- | --- | --- | --- | --- |
| Silt and clay fraction  (<53μm) | Pre-soil | 7.47±0.45a | 15.36±1.07a | 5.32±0.62a | 2.16±0.15a |
|  | FR | 5.96±0.15a | 12.75±1.93a | 4.68±0.31a | 2.70±0.51a |
|  | OM | 5.68±1.59a | 10.00±0.58b | 3.10±0.31b | 1.53±0.67b |
| Micro-aggregates  (53-250μm) | Pre-soil | 3.96±0.11a | 8.13±0.17a | 2.73±0.14a | 0.99±0.14a |
|  | FR | 4.22±0.39a | 8.65±0.70a | 2.79±0.22a | 0.93±0.11a |
|  | OM | 2.55±0.30b | 5.17±0.77b | 1.63±0.24b | 0.60±0.04b |
| Small macro-aggregates  (250-2000μm) | Pre-soil | 8.75±0.07a | 19.01±1.56b | 6.13±0.75a | 2.13±0.52a |
|  | FR | 8.02±0.95a | 19.69±0.60a | 7.16±0.64a | 2.79±0.52a |
|  | OM | 8.63±1.29a | 21.49±0.45a | 7.25±0.80a | 3.03±0.82a |
| Large macro-aggregates (>2000μm) | Pre-soil | 3.78±0.49b | 9.32±0.66c | 3.43±0.22b | 1.32±0.07b |
|  | FR | 4.33±0.30b | 10.57±0.35b | 3.44±0.05b | 1.32±0.09b |
|  | OM | 6.35±0.31a | 16.03±0.47a | 4.95±0.55a | 2.02±0.49a |

The results show means ± standard deviations (n = 3). Different letters at the same columns indicate significant differences (*p* < 0.05) between treatments. FR, chemical fertilizer; OM, organic manure.

(b)

(a)

Fig. S1 Pearson’s correlation matrix between soil chemical properties, SOC chemical components and SOC physical components in microaggregates (a) and macroaggregates (b) fractions. C_mico_, soil organic carbon in microaggregates; N_mico_, total nitrogen in microaggregates; MBC_mico_, microbial biomass carbon in microaggregates; mSC, mineral-incorporated organic carbon in microaggregates; fine iPOC, fine intra-particulate organic carbon in microaggregates; C_maco_, soil organic carbon in macroaggregates; N_maco_, total nitrogen in macroaggregates; MBC_maco_, microbial biomass carbon in macroaggregates; MSC, mineral-incorporated organic carbon in macroaggregates; Fine/coarse iPOC, fine/coarse intra-particulate organic carbon in macroaggregates; A/O-A, alkyl-C/O-alkyl C; AI, aromaticity index; Significant correlation is written in bold and red font at *p* < 0.05.
